# Small Toxic Protein Encoded on Chromosome VII of *Saccharomyces cerevisiae*

**DOI:** 10.1371/journal.pone.0120678

**Published:** 2015-03-17

**Authors:** Koji Makanae, Reiko Kintaka, Koji Ishikawa, Hisao Moriya

**Affiliations:** 1 Research Core for Interdisciplinary Sciences, Okayama University, Kita-ku, Okayama, Japan; 2 Graduate School of Science and Technology, Okayama University, Kita-ku, Okayama, Japan; Louisiana State University Health Sciences Center, UNITED STATES

## Abstract

In a previous study, we found an unknown element that caused growth inhibition after its copy number increased in the 3′ region of *DIE2* in *Saccharomyces cerevisiae*. In this study, we further identified this element and observed that overexpression of a small protein (sORF2) of 57 amino acids encoded in this region caused growth inhibition. The transcriptional response and multicopy suppression of the growth inhibition caused by sORF2 overexpression suggest that sORF2 overexpression inhibits the ergosterol biosynthetic pathway. sORF2 was not required in the normal growth of *S*. *cerevisiae*, and not conserved in related yeast species including *S*. *paradoxus*. Thus, sORF2 (designated as *OTO1*) is an orphan ORF that determines the specificity of this species.

## Introduction

We previously analyzed the copy number limits of most of the protein-coding genes in the budding yeast *Saccharomyces cerevisiae* using the genetic tug-of-war (gTOW) method [1]. In the gTOW method, the copy number of a plasmid containing a target gene (with its native promoter and terminator region) is increased on basis of the selection bias of the *leu2d* gene [2,3]. The copy number of the empty plasmid exceeds 100 in the leucine-negative condition. If the target gene has a copy number limit of <100, the plasmid copy number reflects the copy number limit.

When a target gene has the low copy number limit, we consider that overexpression of the protein encoded by the target gene (i.e., the annotated open reading frame (ORF)) results in growth inhibition. However, elements other than the target gene in the DNA fragment could determine the low copy number. For example, increasing the copy number of a DNA element, overexpression of an RNA element, or overexpression of an unannotated protein could result in growth inhibition.

**Fig 1:**
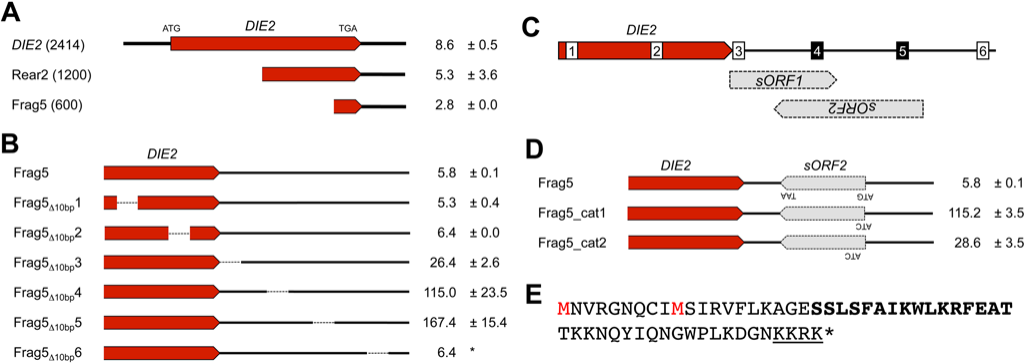
Isolation of the element responsible for the low copy number limit in the *DIE2* region. Copy number limits of DNA fragments from the *DIE2* region. The data were obtained from our previous study [1].Copy number limits of DNA fragments (Frag5 in A) with serial 10-bp deletions every 100 bp. The asterisk indicates that only single experiment was performed.Locations of the small ORFs (*sORF1* and *sORF2*) in the 3′ region of *DIE2*. The numbers indicate the 10-bp deletions analyzed in B. The deletions shown in white did not affect the toxicity of the DNA fragment, whereas the deletion shown in black disrupted the toxicity.Copy number limits of DNA fragments with ATG to ATC substitutions in *sORF2*.Amino acid sequence of *sORF2*. The substituted methionines (ATG codons) in C are shown in red. A potential NLS sequence is underlined, and an amino acid sequence predicted to construct a helical structure is shown in bold letters.

To test this possibility, we previously analyzed the low limit genes by introducing a frameshift mutation to disrupt each annotated ORF and we isolated 10 DNA fragments where frameshift mutations in the annotated ORFs still obtained low copy number limits [1]. We also dissected the fragments and isolated four DNA fragments with unknown elements that determined the low copy number limits. Thus, we isolated a 600-base pair (bp) DNA fragment that contained the 3′ region of *DIE2*, which resulted in a low copy number limit (Frag5 in Fig. 1A) [1].

In this study, we further analyzed this region and showed that expression of a small ORF encoding 58 codons caused growth inhibition.

## Results and Discussion

### Isolation of the element responsible for low copy number limits in the *DIE2* region

To isolate the specific element responsible for the low copy number limit in the 3′ region of *DIE2*, we introduced a series of 10-bp deletions in every 100 bp of Frag5 and measured their copy number limits. As shown in Fig. 1B, deletions of two sites in the downstream region of *DIE2* increased the copy number limit to >100. As shown in Fig. 1C, two small ORFs of >100 bp are encoded in Frag5 (denoted as *sORF1* and *sORF2*). Both of these two 10-bp deletions disrupted *sORF2*, which indicates that *sORF2* might be responsible for the low copy number limit of Frag5.

To disrupt *sORF2* alone, we introduced mutations to change the potential start codons (ATG) of *sORF2* into ATC. The results obtained are shown in Fig. 1D. Frag5 with a mutation that changed the first ATG codon of *sORF2* into ATC possessed a copy number limit of >100. Frag5 with a mutation in the second ATG had a higher limit than the original Frag5, but the limit was still low (28.6 ± 3.5). This result strongly suggests that overexpression of the protein encoded by *sORF2* causes growth inhibition when its copy number is increased in the 3′ region of *DIE2*. Fig. 1E shows the amino acid sequence of *sORF2*.

### High level expression of *sORF2* driven by the *GAL1* promoter inhibits cellular growth

**Fig 2:**
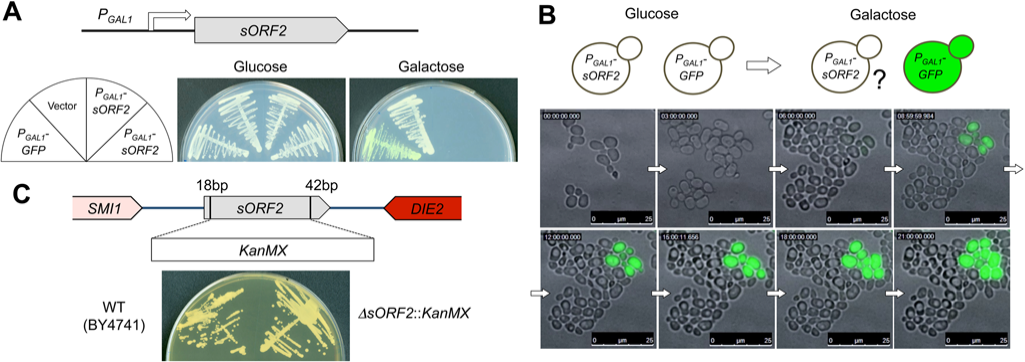
Genetic analyses of sORF2. Overexpression of sORF2 from the *GAL1* promoter (*P*_*GAL1*_). The construct used in this experiment is shown. Cells with pTOW-P_GAL1_-sORF2 (*P*_*GAL1*_ *-sORF2*) were streaked onto SC-glucose and SC-galactose plates. Two independent plasmid clones were analyzed. pTOW40836 (Vector) was used as an empty vector control and pTOW-P_GAL1_-GFP (*P*_*GAL1*_ *-GFP*) was used to monitor the *P*_*GAL1*_ induction.Time-lapse imaging of cells after the induction of sORF2. The cells with pTOW-P_GAL1_-sORF2 (*P*_*GAL1*_ *-ORF2*) and pTOW-P_GAL1_-GFP (*P*_*GAL1*_ *-GFP*) were cultured in SC-glucose mixed at a ratio of 10:1 and then cultivated in SC-galactose medium. *P*_*GAL1*_ *-GFP* was used to monitor the induction of *P*_*GAL1*_. The cellular images shown were obtained every 5min. A movie is available as S1 Movie.Deletion of *sORF2*. The construct used to delete *sORF2* from the chromosome is shown. The strain with *sORF2* deleted was streaked onto a YPD agar plate. The strain BY4741 was used as a wild-type control.

To confirm whether *sORF2* overexpression alone caused growth inhibition, we tried to express *sORF2* from the *GAL1* promoter (*P*_*GAL1*_). As shown in Fig. 2A, yeast cells that harbored the *P*_*GAL1*_ *–sORF2* plasmid did not grow on galactose plates. Next, we observed the growth inhibition process using time-lapse microscopic imaging. As shown in Fig. 2B, at the time point when the induction of *P*_*GAL1*_ *-GFP* was observed, each cell that expressed *sORF2* ceased its proliferation and a large void structure was present. These results indicate that the high level expression of *sORF2* inhibited cellular growth.

### *sORF2* is not required for the normal growth of *S*. *cerevisiae*

To test whether *sORF2* is required for the growth of *S*. *cerevisiae*, we disrupted *sORF2* by replacing it with a kanamycin resistance gene cassette (*KanMX*), as shown in Fig. 2C. The *ΔsORF2*::*KanMX* cells exhibited the same growth as the wild-type cells in normal growth conditions (YPD, 30°C; Fig. 2C).

### Increasing the copy number of the *sORF2*-containing DNA fragment induces the expression of ergosterol synthesis genes

We performed transcriptome analysis (RNAseq) to analyze the cellular response after the overexpression of *sORF2*. We compared the mRNA expression profiles of cells that harbored the vector plasmids and the plasmid containing the *DIE2* 3′ fragment (Rear2, Fig. 1A). Tables 1 and 2 show the genes with significantly different expression levels.

**Table 1:** Genes with higher expression levels in cells that harbored pTOW-Rear2 compared with the control cells.

Name gene	Brief description*
*DAN1*	Cell wall mannoprotein
*DAN4*	Cell wall mannoprotein
*ERG1*	Squalene epoxidase
*ERG3*	C-5 sterol desaturase
*ERG11*	Lanosterol 14-alpha-demethylase
*ERG25*	C-4 methyl sterol oxidase
*EXG1*	Major exo-1,3-beta-glucanase of the cell wall
*FUN30*	Snf2p family member with ATP-dependent chromatin remodeling activity
*MAK16*	Essential nuclear protein
*PBI1*	Putative protein of unknown function
*RNR1*	Major isoform of large subunit of ribonucleotide-diphosphate reductase
*RPA12*	RNA polymerase I subunit A12.2
*SFG1*	Nuclear protein putative transcription factor
*TIR3*	Cell wall mannoprotein
*TIR4*	Cell wall mannoprotein
*TPO2*	Polyamine transporter of the major facilitator superfamily
*YJR005C-A*	Putative protein of unknown function

*Saccharomyces genome database: http://www.yeastgenome.org

**Table 2:** Genes with lower expression levels in cells that harbored pTOW-Rear2 compared with the control cells.

Name gene	Brief description*
*ADH4*	Alcohol dehydrogenase isoenzyme type IV
*ADY2*	Acetate transporter required for normal sporulation
*BTN2*	v-SNARE binding protein
*DSF1*	Putative mannitol dehydrogenase
*ECM23*	Non-essential protein of unconfirmed function
*ENA1*	P-type ATPase sodium pump
*FMP43*	Highly conserved subunit of mitochondrial pyruvate carrier
*FMP45*	Integral membrane protein localized to mitochondria
*HXT6*	High-affinity glucose transporter
*HXT7*	High-affinity glucose transporter
*ISF1*	Serine-rich, hydrophilic protein
*JEN1*	Monocarboxylate/proton symporter of the plasma membrane
*NCE103*	Carbonic anhydrase
*PHO89*	Plasma membrane Na^+^/Pi cotransporter
*PUT1*	Proline oxidase
*RGI2*	Protein of unknown function
*SMA1*	Protein of unknown function involved in prospore membrane assembly
*SPG1*	Protein required for high temperature survival during stationary phase
*SPG4*	Protein required for high temperature survival during stationary phase
*SPL2*	Protein with similarity to cyclin-dependent kinase inhibitors
*TMA10*	Protein of unknown function that associates with ribosomes
*YBR285W*	Putative protein of unknown function
*YGR067C*	Putative protein of unknown function
*YLR307C-A*	Putative protein of unknown function
*YNL194C*	Integral membrane protein
*YNL195C*	Protein of unknown function

*Saccharomyces genome database: http://www.yeastgenome.org

We analyzed the enriched genes based on gene ontology (GO) terms. The genes with higher expression levels in the cells that harbored the pTOW-Rear2 plasmid were significantly enriched in terms of genes involved in the ergosterol biosynthesis pathway (*p* = 2.2e^−4^). Eight genes (*DAN1*, *DAN4*, *ERG1*, *ERG3*, *ERG11*, *ERG25*, *TIR3*, and *TIR4*) with higher expression levels were identified as genes that could be induced by treatment with ketoconazole [4]. Ketoconazole is known to inhibit the ergosterol biosynthetic pathway [5]; thus, *sORF2* overexpression appeared to affect this pathway. The genes with lower expression levels were not significantly enriched with respect to GO terms. They however contained many genes encoding transporters and membrane proteins, such as *ADY2*, *ENA1*, *FMP43*, *FMP45*, *HXT6*, *HXT7*, *JEN1*, *PHO89*, *SMA1*, and *YNL194C*, suggesting that *sORF2* overexpression modulates the expression of membrane proteins.

### Expression analysis of *sORF2*

**Fig 3:**
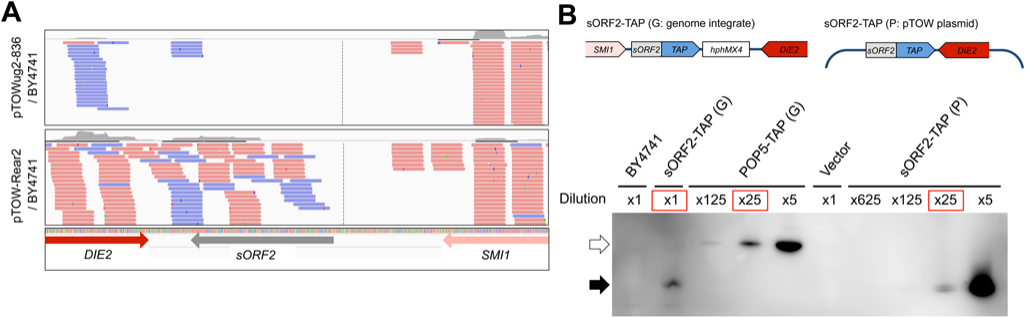
Expression analysis of *sORF2*. RNAseq analysis of the *sORF2* region of the strain BY4741 with the control vector (pTOWug2–836) and pTOW-Rear2. Parts of the detected reads are shown. The locations of *DIE2*, *sORF2*, and *SMI1* are indicated.Western blot analysis of sORF2 using TAPtag. Expression of sORF2-TAP from the genomic region or plasmids was detected using peroxidase anti-peroxidase soluble complex. BY4714 is a negative control strain without any TAP-tagged protein expressed. Vector is another negative control, in which BY4741 harbors an empty vector (pTOWug2–836). Cells of BY4741, sORF2-TAP (genome), and POP5-TAP (genome) were cultivated in YPD medium; cells of Vector and sORF2-TAP (plasmid) were cultivated in SC—Ura medium. Dilution indicates the fold-dilution of the cellular lysate applied to the gel. Red-squared dilutions were used to calculate the expression levels of TAP-tagged proteins. The white arrowhead indicates the expected molecular weight of Pop5-TAP protein (39.6kDa), and the black arrowhead indicates the one of sORF2-TAP (27.1 kDa). Structures of sORF2-TAP constructs are shown.

We analyzed the RNAseq data to determine whether *sORF2* is transcribed. As shown in Fig. 3A, transcript reads containing *sORF2* were not detected in the mRNAs from BY4741 that harbored an empty vector pTOWug2–836, whereas a large number of transcript reads were detected in the mRNAs that harbored pTOW-Rear2.

To test whether *sORF2* was translated, we attached the tandem affinity purification (TAP) tag to *sORF2* and attempted to detect the TAP-tagged *sORF2* by Western blotting. As shown in Fig. 3B, sORF2-TAP expressed from its genomic region was detected, and the expression of sORF2-TAP from the plasmid was highly increased. The expression of sORF2 from its genomic region was detected in the log phage cell lysate, but not in the post-log phase lysate (S1 Fig.). The expression was not increased under mating conditions (S1 Fig.).

We further estimated the expression level of sORF2-TAP in comparison to the expression level of a reference protein Pop5-TAP, whose protein copy number was previously determined (2230 copies/cell) [6]. As the result, the expression level of sORF2-TAP from its genomic region was estimated to be 45 copies/cell, which corresponds to the level of lowly expressed proteins [6]. The estimated expression level of sORF2-TAP from the plasmid was 1938 copies/cell. It should be noted that the copy number limit of the plasmid that contained the *sORF2-TAP* DNA fragment was >100 (data not shown). This suggests that the small size of sORF2 itself is required to inhibit growth.

**Fig 4:**
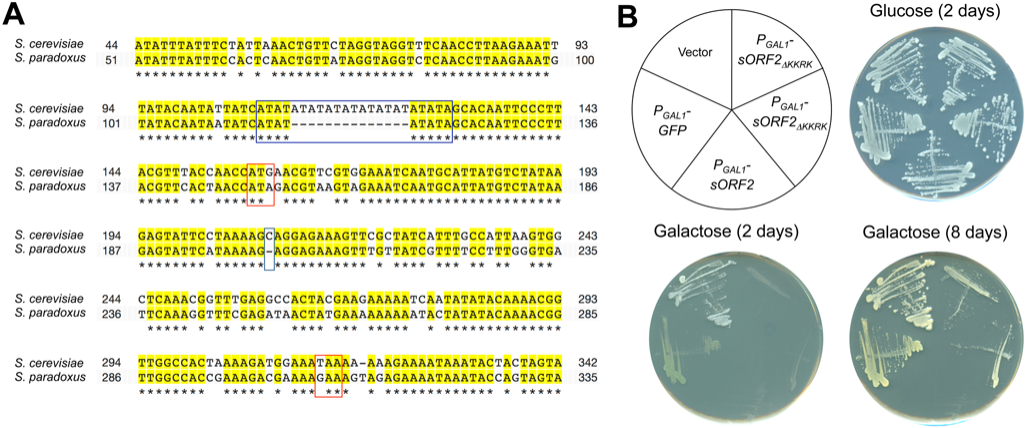
Structural analysis of sORF2. Alignment of the *sORF2* regions of *S*. *cerevisiae* and *S*. *paradoxus*. Identical nucleotides are shown in yellow. ATG and STOP codons of *sORF2* are shown in red. A TATA repeat and deletion in the *S*. *paradoxus* sequence are indicated in blue. The image is a snapshot from the fungal sequence alignment of SGD (http://www.yeastgenome.org/cache/fungi/YGR229C.html). The nucleotide numbers indicate the positions relative to the stop codon of *SMI1*.Overexpression of sORF2 without the potential NLS (sORF2 _ΔKKRK_). The construct used in this experiment is shown. Cells with pTOW-P_GAL1_-sORF2 (*P*_*GAL1*_ *-sORF2*) or pTOW-P_GAL1_-sORF2_ΔKKRK_ (*P*_*GAL1*_ *-sORF2*_*ΔKKRK*_) were streaked onto SC-glucose and SC-galactose plates and incubated for indicated days. pTOW40836 (Vector) was used as an empty vector control and pTOW-P_GAL1_-GFP (*P*_*GAL1*_ *-GFP*) was used to monitor the *P*_*GAL1*_ induction.

Currently, we do not know the reason why we could not detect the mRNA of *sORF2* expressed from its genomic region by our RNAseq analysis above. Although it is possible that integrating TAP-tag sequence and a marker gene stimulated the expression of *sORF2*, the result still suggests that there is an expression potential from the *sORF2* locus. Supporting this idea, there is a TA repeat in the upstream region of *sORF2*, which provides potential binding sites for transcriptional factors such as the TATA-binding protein Spt15 (S2 Fig.). Notably, the TA repeat is far shorter in the corresponding genomic region of *S*. *paradoxus*, which lacks *sORF2* (Fig. 4A). These binding sites might function as promoters for *sORF2*.

### Multicopy *UBP7* and *PRM1* suppress the growth inhibition caused by the high copy number *sORF2*-containing DNA fragment

**Fig 5:**
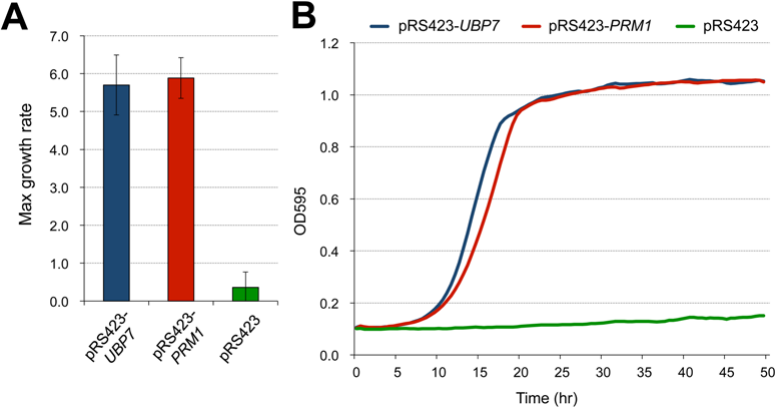
Multicopy suppressors of growth inhibition after increasing the copy number in the *DIE2* 3′ fragment. Maximum growth rate of BY4741 cells that harbored both pTOW-Rear2 and the suppressor plasmids (pRS423-*UBP7* and pRS423-*PRM1*, and the empty vector, pRS423) in SC—Ura—His medium. The averages and standard deviations from six independent experiments are shown.Growth curves of the BY4741 cells that harbored both pTOW-Rear2 and the suppressor plasmids in SC—Ura—His medium. One representative data is shown from each experiment.

To further elucidate the molecular mechanism responsible for growth inhibition by *sORF2*, we attempted to isolate multicopy suppressors of the growth inhibition caused by high copy number pTOW-Rear2. As shown in Fig. 5, we isolated two multicopy suppressors, *UBP7* and *PRM1*. *UBP7* encodes a ubiquitin protease (UBPs) that controls protein degradation [7]. *PRM1* encodes a pheromone-regulated membrane protein, which is involved in membrane fusion during mating [8]. *PRM1* is known to have a genetic interaction with *ERG* genes [9,10]. This result also suggests the involvement of *sORF2* in the ergosterol biosynthetic pathway.

### Structural analysis of sORF2

In order to speculate the molecular function of sORF2, we performed some bioinformatics analyses. We first performed the BLAST search toward the protein sequences stored at NCBI database (http://blast.ncbi.nlm.nih.gov/), but we could not obtain any significantly similar protein.

The corresponding ORF was not conserved in any closely-related yeast species (*S*. *paradoxus*, *S*. *bayanus*, *S*. *mikatae*, *S*. *castellii*, and *S*. *kudriavzevii*). Fig. 4A shows the comparison of the corresponding genomic locus from *S*. *cerevisiae* and *S*. *paradoxus* (most closely-related species to *S*. *cerevisae*), as an example.

During the structural analysis, we noticed that sORF2 contained a consensus sequence of nuclear localization signals (K-K/R-X-K/R) [11] at its C-terminal (underlined in Fig. 1E). To test if this potential nuclear localization signal (NLS) is important for the toxicity of sORF2, we overexpressed sORF2 without the sequence (sORF2_ΔKKRK_). As shown in Fig. 4B, yeast cells that harbored the *P*_*GAL1*_ *–sORF2*_*ΔKKRK*_ plasmid grew on galactose plates, but much slower than the cells with the empty vector or *P*_*GAL1*_ *–GFP* plasmids. This result indicates that the potential NLS is partly required (but not essential) for the toxicity of sORF2.

We next tried to predict the secondary and tertiary structure of sORF2 using a protein homology/analogy recognition engine, Phyre2 (http://www.sbg.bio.ic.ac.uk/phyre2/). The analysis predicted that there was a helical structure in the middle of the protein (shown in bold letters in Fig. 1E) based on its similarity with two template proteins (d1k78a1 and d6paxa1) with the confidence scores > 70 (the prediction results are summarized in S3 Fig.). Because the template proteins were both structurally classified into DNA/RNA-binding 3-helical bundle (Fold), homeodomain-like (superfamily), and paired domain (family), sORF2 might have DNA/RNA binding activity.

### sORF2 (OTO1/YGR228C-A) as an orphan ORF

In this study, we obtained evidence that overexpression of a small ORF of 58 codons (*sORF2*) encoded within the 3′ region of *DIE2* causes growth inhibition. Our results also suggest that sORF2 overexpression affects the ergosterol synthetic pathway. Based on the fact that sORF2 has a potential NLS and a helical structure involved in DNA/RNA binding, sORF2 might function through its nuclear function such as transcriptional regulation.

*sORF2* was not identified in previous studies that aimed to detect small ORFs based on their expression and evolutionary conservation [12–15]. In fact, *sORF2* is not conserved in the corresponding genomic region of the most closely-related yeast species *S*. *paradoxus* (Fig. 4A). We thus think that *sORF2* is an orphan ORF (ORFan) [16, 17], which distinguishes species by functioning in species-specific cellular situations, and propose its name as *OTO1* (ORFan toxic when overexpressed) with its locus name *YGR228C-A*.

Our gTOW approach might be useful for isolating other ORFans. In fact, we had isolated three more genomic loci potentially contain unannotated toxic elements when the copy numbers were increased [1].

## Materials and Methods

### Strains and growth conditions

BY4741 (*MATa his3Δ0 leu2 Δ0 met15 Δ0 ura3 Δ0*) [18] was used as the host yeast strain to test the toxicity of *DIE2* fragments and s*ORF2*. The *sORF2* deletion strain was created, as follows: The genomic region of *sORF2* (from ATG to stop) in BY4743 (*MAT*a/α *his3* Δ*1/his3* Δ*1 leu2* Δ*0/leu2* Δ*0 LYS2/lys2* Δ*0 met15* Δ*0/MET15 ura3* Δ*0/ura3* Δ*0*) [18] was replaced by the *KanMX6* cassette using a DNA fragment, which was amplified by PCR with the primers OHM0969 and OHM0970 using pKT127 [19] as a template. The strains were sporulated, and the tetrads were dissected. After genotypic analysis of the tetrads, haploid deletion strains were isolated. The *sORF2-TAP* strain was created, as follows: A *sORF2-TAP* fragment was amplified by PCR with the primers OHM1030 and OHM1032 using pTOW-sORF2-TAP. A *hphMX4* fragment was amplified by PCR with primers the OHM1031 and OH1033 using pAG34 [20]. Both fragments were introduced into BY4741 to integrate *sORF2-TAP-hphMX4* into the genomic region of *sORF2*. BY4742 (*MAT*α *his3*Δ*1 leu2*Δ*0 lys2*Δ*0 ura3*Δ*0*) [18] was used for a mating partner of BY4741 with *sORF2-TAP-hphMX4*.

Yeast cells were grown in standard growth conditions [21]. The PCR primers used to amplify the DNA fragments employed in strain construction are listed in S1 Table.

### Plasmids used in this study

The plasmids used in this study are listed in Table 3. The plasmids were constructed on the basis of the homologous recombination activity of yeast cells [22]. The PCR primers used to amplify the DNA fragments, which were employed in plasmid construction are listed in S1 Table.

**Table 3:** Plasmids used in this study.

Name	Description	Source
pTOWug2–836	*Amp* ^*R*^, *ColE1ori*, *2μori*, *URA3-yEGFP*, *leu2d*	[1]
pTOW40836	*Amp* ^*R*^, *ColE1ori*, *2μori*, *URA3*, *leu2d*	[3]
pTOW-Rear2	*DIE2* 3′ region (Rear2) cloned into pTOWug2–836	[1]
pTOW-Frag5	*DIE2* 3′ region (Frag5) cloned into pTOWug2–836	[1]
pTOW-Frag5_Δ10bp_1	pTOW-Frag5 containing 10-bp deletion (16–25)	This study
pTOW-Frag5_Δ10bp_2	pTOW-Frag5 containing 10-bp deletion (116–125)	This study
pTOW-Frag5_Δ10bp_3	pTOW-Frag5 containing 10-bp deletion (216–225)	This study
pTOW-Frag5_Δ10bp_4	pTOW-Frag5 containing 10-bp deletion (312–321)	This study
pTOW-Frag5_Δ10bp_5	pTOW-Frag5 containing 10-bp deletion (416–425)	This study
pTOW-Frag5_Δ10bp_6	pTOW-Frag5 containing 10-bp deletion (516–525)	This study
pTOW-Frag5_cat1	First ATG of sORF2 changed into ATC in pTOW-Frag5	This study
pTOW-Frag5_cat2	Second ATG of sORF2 changed into ATC in pTOW-Frag5	This study
pTOW-P_GAL1_-GFP	*P*_*GAL1*_ *-yEGFP-T*_*GAL1*_ cloned into pTOW40836	This study
pTOW-P_GAL1_-sORF2	*P*_*GAL1*_ *-sORF2-T*_*GAL1*_ cloned into pTOW40836	This study
pTOW-P_GAL1_-sORF2 _ΔKKRK_	*P*_*GAL1*_ *-sORF2*_*ΔKKRK*_ *-T*_*GAL1*_ cloned into pTOW40836	This study
pTOW-sORF2-TAP	TAPtag inserted into the C-terminal of sORF2 of pTOW-Frag5	This study
pRS423ks	*Amp* ^*R*^, *ColE1ori*, *2μori*, *HIS3*	[1]
pRS423-*UBP7*	*UBP7* cloned into pRS423ks	This study
pRS423-*PRM1*	*PRM1* cloned into pRS423ks	This study

### Measurement of the plasmid copy number limit

The copy number limits of plasmids were measured as described in our previous study [1]. Briefly, DNA from yeast cells grown in SC—Ura, SC—Ura—Leu, or SC—Ura—His medium were extracted, and the relative plasmid copy number compared with the genomic DNA in the DNA solution was measured using real-time PCR. *HIS3*, *LEU2*, and *LEU3* genes were detected as indicators of the plasmid copy number for pRS423ks, pTOWug2–836/40836, and genomic DNA, respectively. More than two independent experiments were performed for each experiment otherwise stated.

### RNAseq analysis

Yeast cells that harbored pTOWug2–836, pTOW40836, and pTOW-Rear2 were cultivated in SC—Ura medium until the mid-log phase, and RNA from each culture was then isolated using the hot phenol method [23]. A cDNA library was prepared using a SureSelect strand-specific RNA library preparation kit (G9691A, Agilent), and sequencing was performed using an Illumina Hiseq2500 with TruSeq SBS kit v3-HS. The software connected to GenomeSpace (http://www.genomespace.org) was used for the sequence data analysis. The sequence data were analyzed using TopHat (ver. 6) and Cufflink/cuffdiff (ver.4) on the GenePattern platform (http://genepattern.broadinstitute.org), with sacCer3 for gene annotation (http://genome.ucsc.edu/cgi-bin/hgTables). First, we isolated genes that differed significantly (FDR < 0.5) between pTOWug2–836 and pTOW-Rear2 (Comp1), pTOW40836 and pTOW-Rear2 (Comp2), pTOWug2–836 and pTOW40836 (Comp3), and the pTOW-Rear2 duplicates (Comp4). Next, we prepared a gene list from genes isolated in Comp1 or Comp2, but not in Comp3 or Comp4 (summarized in S4 Fig. and S2 Table). The Integrative Genomics Viewer (IGV2.3, http://www.broadinstitute.org/igv/) was used to visualize the sequence reads shown in Fig. 3A. The GO, publication, and pathway enrichments were analyzed using YeastMine (http://yeastmine.yeastgenome.org).

### Microscopic observation

Cells were cultivated in SC—Ura medium until the mid-log phase and the cells were then transferred to SC-galactose—Ura medium, before being applied to a PDMS microfluidic chamber (YC-1, Warner instruments). Cellular images were acquired every 5 min using a Leica DM6000 B microscope. GFP fluorescence was determined using a GFP filter cube (excitation filter 470/40 and emission filter 525/50).

### Western blot analysis

Western blotting was performed as described previously [24]. Briefly, proteins extracted from the 0.25 OD_600_ cells (with indicated fold dilutions) cultivated in the indicated medium were separated by SDS-PAGE and transferred onto a PVDF membrane. The TAP-tagged protein was then detected using peroxidase anti-peroxidase soluble complex (P1901l, Sigma-Aldrich). The chemiluminescent image was taken and the intensity of each band was measured using the LAS-4000 image analyzer (GE Healthcare).

### Multicopy suppressor screening

A multicopy plasmid library where most of the genes in *S*. *cerevisiae* were cloned into pRS423ks (our laboratory stock) was introduced into yeast strains that harbored pTOW-Rear2. Next, the colonies were grown on SC—Ura—His plates and then replica-plated onto SC—Ura—Leu—His plates. The plasmids were recovered from the colonies grown on SC—Ura—Leu—His and the DNA sequences of inserts in the plasmids were determined. The suppressor activities of the isolated candidates were re-evaluated by measuring the growth of the cells that harbored both pTOW-Rear2 and the suppressor plasmids in SC—Ura—His medium. Cellular growth was measured by monitoring OD_595_ every 30 min using a microplate reader (Infinite F200, TECAN). The maximum growth rate was calculated as described previously [2, 3].

## Supporting Information

S1 Table Oligo DNA primers used in this study. (XLSX)

S2 Table Genes isolated by the RNAseq analysis. (XLSX)

S1 Fig Expressions of sORF2-TAP from its genomic region in different conditions. Expression of sORF2-TAP from the genomic region under indicated conditions were detected using peroxidase anti-peroxidase soluble complex. Cellular lysates from the 0.0625 OD_600_ cells were loaded. To create mating conditions, BY4741 with *sORF2-TAP-hphMX4* cells were mixed with BY4742 cells on a YPD agar plate and incubated for 2 hours in prior to prepare of the cellular lysate. BY4741 with *sORF2-TAP-hphMX4* cells were cultivated in YPD medium to prepare log phase cells and post-log phase cells. The cellular density of the cultures were shown as OD_600_.
(TIF)

S2 Fig Potential transcription factor binding sites located upstream of *sORF2*. The image is a snapshot from the YeTFaSCo analysis (http://yetfasco.ccbr.utoronto.ca). The arrowhead indicates sORF2.
(TIF)

S3 Fig Predicted helical structure in sORF2. The 3D structures, summaries, and alignments are shown. The images were snapshots of displayed on the Phyre2 website (http://www.sbg.bio.ic.ac.uk/phyre2/).
(TIF)

S4 Fig Isolation of genes whose expressions were significantly changed upon increase in DIE2-Rear2 fragment. We first isolated genes showing significant difference (FDR < 0.5) between; pTOWug2–836 and pTOWug2-Rear2 (Comp1), pTOW40836 and pTOWug2-Rear2 (Comp2), pTOWug2–836 and pTOW40836 (Comp3), and between pTOWug2-Rear2 duplicates (Comp4). We then made a gene list, which contained true positives, from isolated genes in Comp1 or Comp2, but neither in Comp3 nor Comp4 (see the Venn diagram).
(TIF)

S1 Movie Time-lapse movie of cells after the induction of sORF2. (MOV)
