# Supplementary figures and images for: Small Toxic Protein Encoded on Chromosome VII of Saccharomyces cerevisiae
Source: PLoS One. 2015 Mar 17;10(3):e0120678. doi: 10.1371/journal.pone.0120678 (PMC4363601; doi:10.1371/journal.pone.0120678)

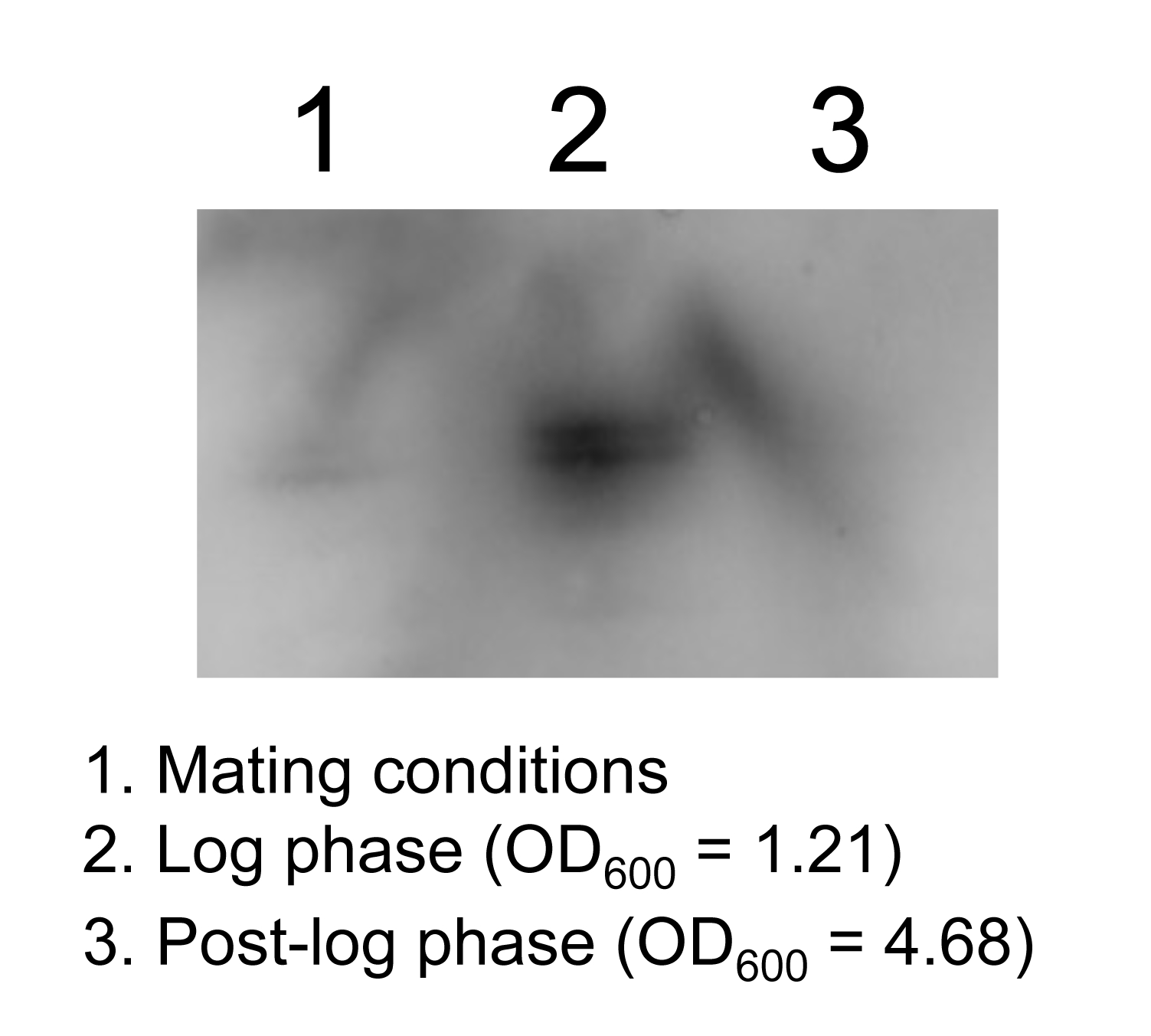

Supplement: S1 Fig — Expression of sORF2-TAP from the genomic region under indicated conditions were detected using peroxidase anti-peroxidase soluble complex. Cellular lysates from the 0.0625 OD600 cells were loaded. To create mating conditions, BY4741 with sORF2-TAP-hphMX4 cells were mixed with BY4742 cells on a YPD agar plate and incubated for 2 hours in prior to prepare of the cellular lysate. BY4741 with sORF2-TAP-hphMX4 cells were cultivated in YPD medium to prepare log phase cells and post-log phase cells. The cellular density of the cultures were shown as OD600. (TIF) [file pone.0120678.s003.tif]

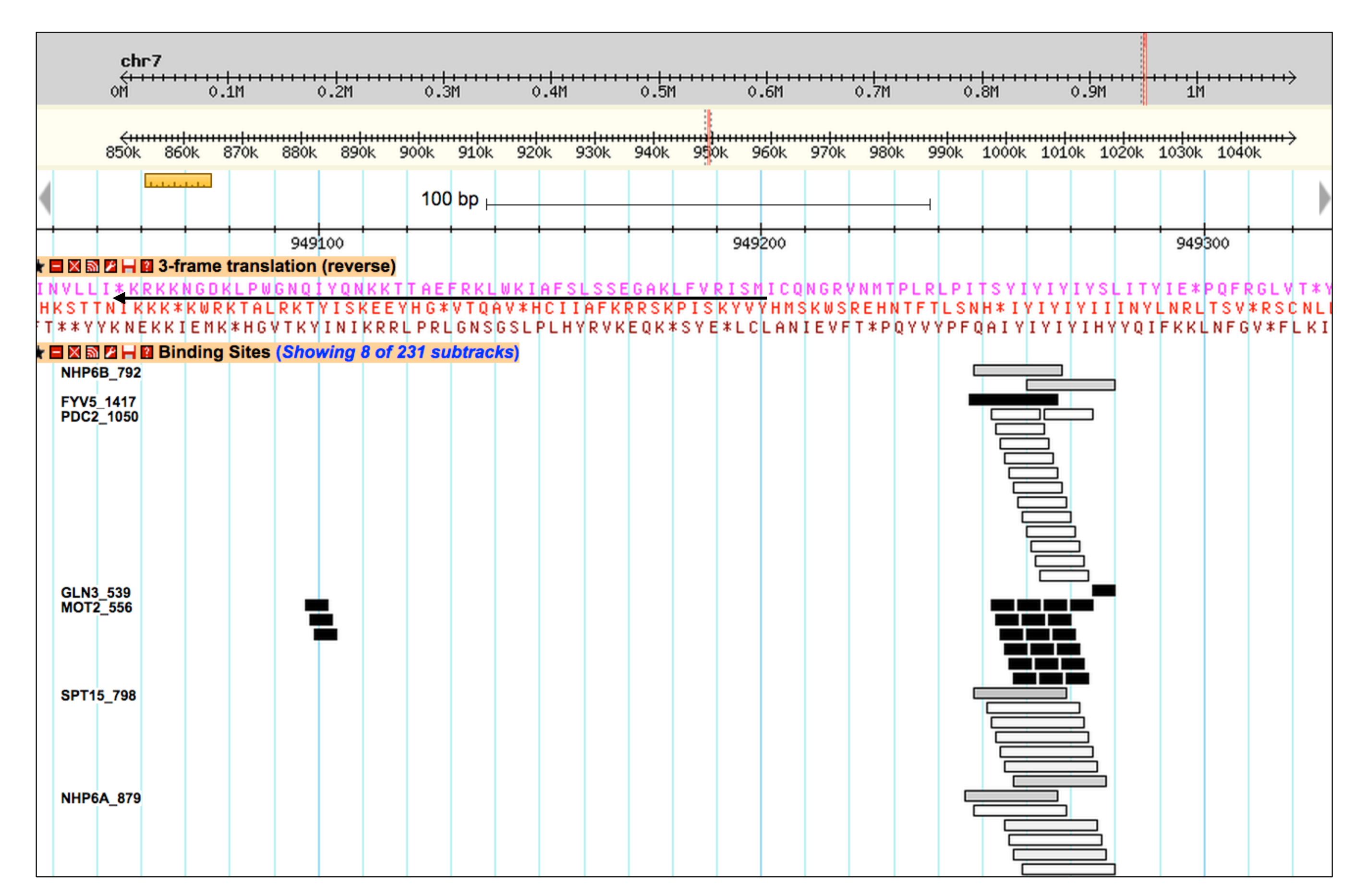

Supplement: S2 Fig — The image is a snapshot from the YeTFaSCo analysis (http://yetfasco.ccbr.utoronto.ca). The arrowhead indicates sORF2. (TIF) [file pone.0120678.s004.tif]

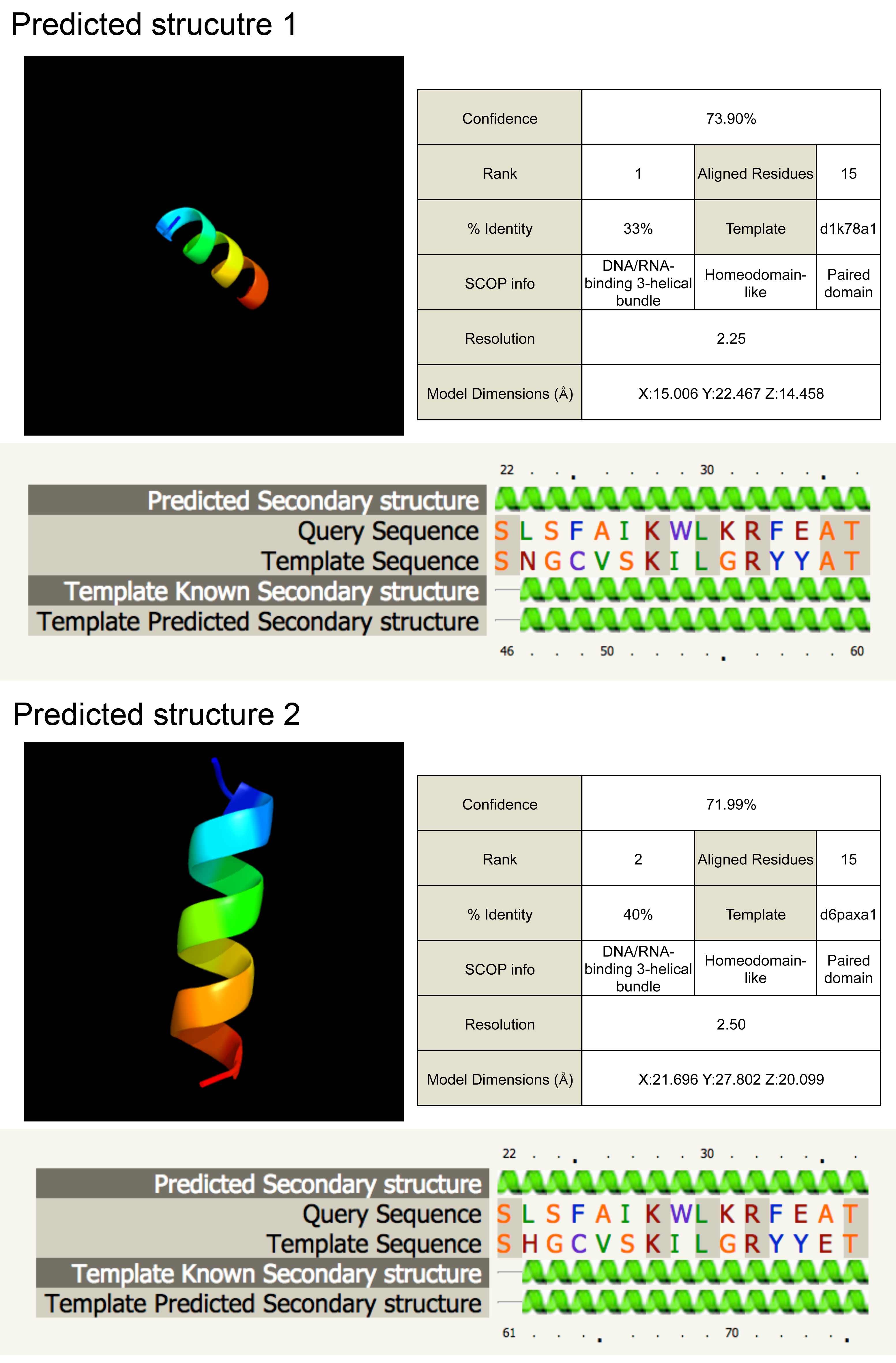

Supplement: S3 Fig — The 3D structures, summaries, and alignments are shown. The images were snapshots of displayed on the Phyre2 website (http://www.sbg.bio.ic.ac.uk/phyre2/). (TIF) [file pone.0120678.s005.tif]

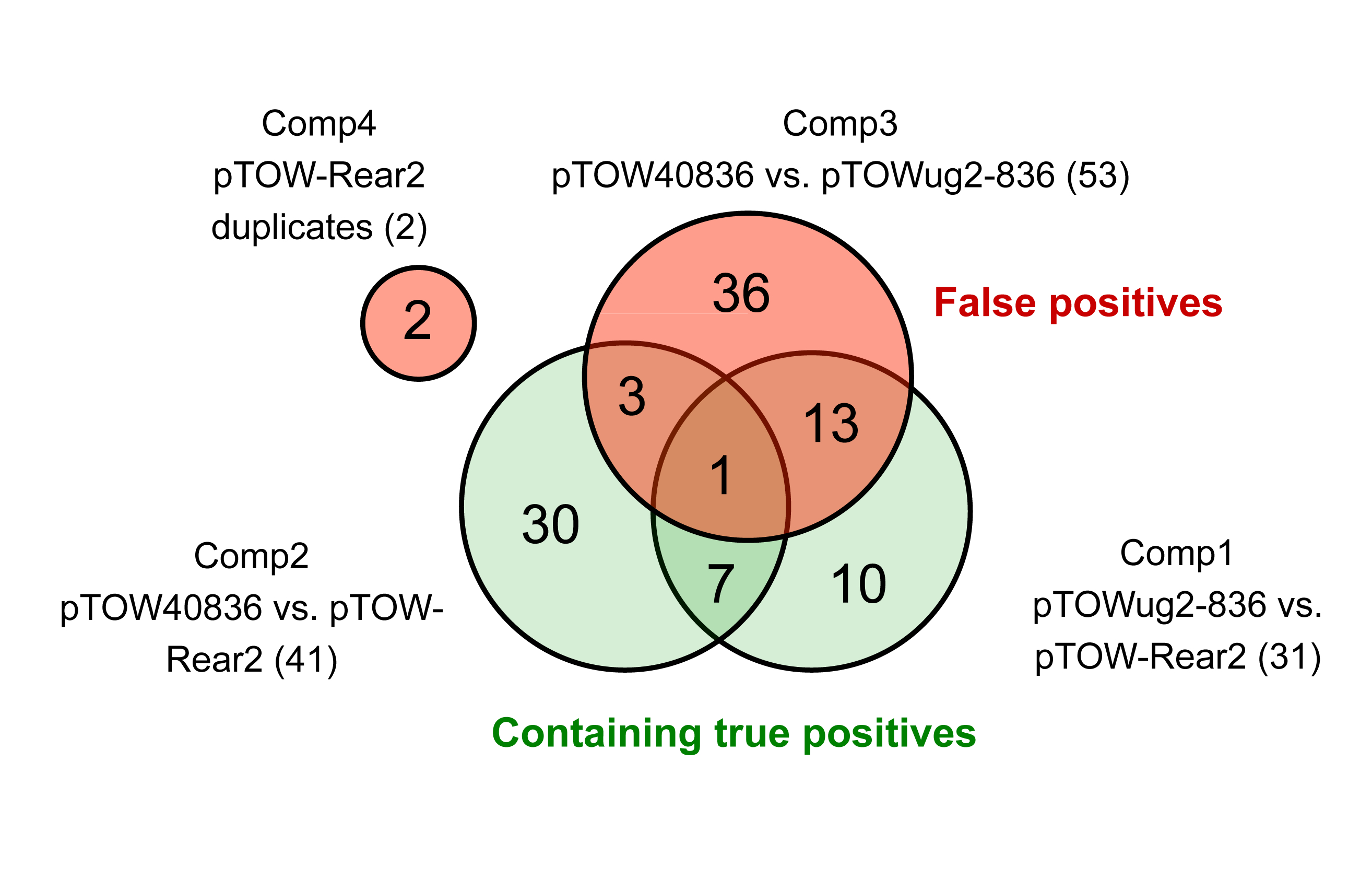

Supplement: S4 Fig — We first isolated genes showing significant difference (FDR < 0.5) between; pTOWug2–836 and pTOWug2-Rear2 (Comp1), pTOW40836 and pTOWug2-Rear2 (Comp2), pTOWug2–836 and pTOW40836 (Comp3), and between pTOWug2-Rear2 duplicates (Comp4). We then made a gene list, which contained true positives, from isolated genes in Comp1 or Comp2, but neither in Comp3 nor Comp4 (see the Venn diagram). (TIF) [file pone.0120678.s006.tif]
